# Factors associated with circulatory death after out-of-hospital cardiac arrest: a population-based cluster analysis

**DOI:** 10.1186/s13613-023-01143-8

**Published:** 2023-06-09

**Authors:** Yannick Binois, Marie Renaudier, Florence Dumas, Younès Youssfi, Frankie Beganton, Daniel Jost, Lionel Lamhaut, Eloi Marijon, Xavier Jouven, Alain Cariou, Wulfran Bougouin, the Sudden-Death-Expertise-Center investigators, F. Adnet, J. M. Agostinucci, N. Aissaoui-Balanant, V. Algalarrondo, F. Alla, C. Alonso, W. Amara, D. Annane, C. Antoine, P. Aubry, E. Azoulay, F. Beganton, C. Billon, W. Bougouin, J. Boutet, C. Bruel, P. Bruneval, A. Cariou, P. Carli, E. Casalino, C. Cerf, A. Chaib, B. Cholley, Y. Cohen, A. Combes, J. M. Coulaud, M Crahes, D. Da Silva, V. Das, A. Demoule, I. Denjoy, N. Deye, J. L. Diehl, S. Dinanian, L. Domanski, D. Dreyfuss, D. Duboc, J. L. Dubois-Rande, F. Dumas, J. Duranteau, J. P. Empana, F. Extramiana, J. Y. Fagon, M. Fartoukh, F. Fieux, M. Gabbas, E. Gandjbakhch, G. Geri, B. Guidet, F. Halimi, P. Henry, F. Hidden Lucet, P. Jabre, L. Joseph, D. Jost, X. Jouven, N. Karam, H. Kassim, J. Lacotte, K. Lahlou-Laforet, L. Lamhaut, A. Lanceleur, O. Langeron, T. Lavergne, E. Lecarpentier, A. Leenhardt, N. Lellouche, V. Lemiale, F. Lemoine, F. Linval, T. Loeb, B. Ludes, C. E. Luyt, A. Maltret, N. Mansencal, N. Mansouri, E. Marijon, J. Marty, E. Maury, V. Maxime, B. Megarbane, A. Mekontso-Dessap, H. Mentec, J. P. Mira, X. Monnet, K. Narayanan, N. Ngoyi, M. C. Perier, O. Piot, R. Pirracchio, P. Plaisance, B. Plaud, I. Plu, J. H. Raphalen, M. Raux, F. Revaux, J. D. Ricard, C. Richard, B. Riou, F. Roussin, F. Santoli, F. Schortgen, A. Sharifzadehgan, T. Sharshar, G. Sideris, T. Similowski, C. Spaulding, J. L. Teboul, J. F. Timsit, J. P. Tourtier, P. Tuppin, C. Ursat, O. Varenne, A. Vieillard-Baron, S. Voicu, K. Wahbi, V. Waldmann

**Affiliations:** 1 Université de Paris, INSERM U970, Paris Cardiovascular Research Center (PARCC), European Georges Pompidou Hospital, 75015 Paris, France; 2 Medical Intensive Care Unit, AP-HP, Cochin Hospital, 75014 Paris, France; 3 Paris Sudden Death Expertise Center, 75015 Paris, France; 4 Emergency Department, AP-HP, Cochin-Hotel-Dieu Hospital, 75014 Paris, France; 5 Cardiology Department, AP-HP, European Georges Pompidou Hospital, 75015 Paris, France; 6 BSPP (Paris Fire-Brigade Emergency-Medicine Department), 1 Place Jules Renard, 75017 Paris, France; 7 Intensive Care Unit and SAMU 75, Necker Enfants-Malades Hospital, 75014 Paris, France; 8 Medical Intensive Care Unit, Ramsay Générale de Santé, Hôpital Privé Jacques Cartier, 6 Avenue du Noyer Lambert, 91300 Massy, France; 9 Center for Research in Economics and Statistics, 91120 Palaiseau, France; 10 AfterROSC network, Paris, France

**Keywords:** Sudden death, Mode of death, Shock, Post-resuscitation, Personalized medicine

## Abstract

**Background:**

Out-of-hospital cardiac arrest (OHCA) is a common cause of death. Early circulatory failure is the most common reason for death within the first 48 h. This study in intensive care unit (ICU) patients with OHCA was designed to identify and characterize clusters based on clinical features and to determine the frequency of death from refractory postresuscitation shock (RPRS) in each cluster.

**Methods:**

We retrospectively identified adults admitted alive to ICUs after OHCA in 2011–2018 and recorded in a prospective registry for the Paris region (France). We identified patient clusters by performing an unsupervised hierarchical cluster analysis (without mode of death among the variables) based on Utstein clinical and laboratory variables. For each cluster, we estimated the hazard ratio (HRs) for RPRS.

**Results:**

Of the 4445 included patients, 1468 (33%) were discharged alive from the ICU and 2977 (67%) died in the ICU. We identified four clusters: initial shockable rhythm with short low-flow time (cluster 1), initial non-shockable rhythm with usual absence of ST-segment elevation (cluster 2), initial non-shockable rhythm with long no-flow time (cluster 3), and long low-flow time with high epinephrine dose (cluster 4). RPRS was significantly associated with this last cluster (HR, 5.51; 95% confidence interval 4.51–6.74).

**Conclusions:**

We identified patient clusters based on Utstein criteria, and one cluster was strongly associated with RPRS. This result may help to make decisions about using specific treatments after OHCA.

**Supplementary Information:**

The online version contains supplementary material available at 10.1186/s13613-023-01143-8.

## Introduction

Out-of-hospital cardiac arrest (OHCA) is a common cause of death, with an annual incidence of 46 000 cases in France [1] and over 300 000 cases in the US. Despite decades of research, the prognosis remains poor, with less than 10% of patients surviving to hospital discharge [2, 3]. Most patients die before hospital admission, and among patients admitted alive, about 70% die in the intensive care unit (ICU) [2].

Death in the ICU may occur due to refractory postresuscitation shock (RPRS), or to hypoxic–ischemic brain injury [brain death or withdrawal of life-sustaining treatments (WLST)] [4–6]. Tailoring the treatment strategy to the most likely mechanism of death might improve outcomes. Interventions specifically designed to prevent death from RPRS include steroids [7–10], ciclosporine [11], extracorporeal support [12, 13], and goal-directed hemodynamic optimization [14–16]. Until now, trials testing these interventions have failed to show benefits. However, considering the heterogeneity of OHCA, specific interventions could have variable effects (both in magnitude and direction of treatment effect), also known as heterogeneity of treatment effect. To address this issue in other heterogenous syndromes, identification of homogenous clusters has been proposed to personalize treatment (in acute respiratory distress syndrome [17], or sepsis [18]), to offer the right therapy to the right patient. Accordingly, recent guidelines indicate that the treatment of OHCA should target goals determined on a case-by-case basis [19]. Contrary to this recommendation, the above-listed trials included unselected patients, most of whom died of events other than RPRS. Patient selection for specific treatment would require the identification of factors associated with death from RPRS. This goal could be achieved by using clustering techniques to reveal commonalities and identify uniform patient profiles within a heterogenous population.

The objective of this unsupervised clustering analysis of data from a prospectively established population-based registry was to identify patient subgroups with similar baseline features then to determine whether any of these subgroups was at particularly high risk for RPRS.

## Methods

This study is reported according to strengthening the Reporting of Observational studies in Epidemiology guidelines [20]. We performed a population-based observational study, with a retrospective unsupervised clustering analysis of prospectively collected data from a multicentric cohort in France, between May 15, 2011, and December 31, 2018.

### Population

In Paris and its inner suburbs, which have a population of about 6.8·million, patients with OHCA are managed on-scene by mobile emergency units and fire departments. Those who achieve the return of spontaneous circulation (ROSC) are taken to an ICU in a tertiary hospital. Since May 2011, these patients, if older than 18 years, are recorded in a prospective multicenter population-based registry managed by the Paris-Sudden Death Expertise Center [2, 21, 22]. The appropriate ethics committees approved the registry (CNIL approval #912309 and CCTIRS approval #12336).

We retrospectively studied the data recorded in the registry between May 15, 2011, and December 31, 2018. We included only patients with OHCA due to cardiac causes. We did not include patients with OHCA due to external factors (e.g., trauma, overdose, or drowning) [23], patients for whom no identifying data were available (unknown patients), or patients whose reason for death was unknown or unclassifiable according to Witten et al.[6] In order to be representative, we included all patients with OHCA due to cardiac causes recorded in this registry, consecutively and without selection. No sample size calculation was performed.

### Data collection

Data were collected prospectively in the registry according to Utstein criteria [24], including sex, age, presence of a witness, cardiopulmonary resuscitation (CPR) performed by a bystander, location of the OHCA (home vs. public place), first-recorded cardiac rhythm, total epinephrine dose delivered by emergency medical staff during advanced life support, no-flow time (time from collapse to the initiation of CPR) and low-flow time (time from the initiation of CPR to the ROSC), targeted temperature management, arterial lactate and serum creatinine at ICU admission, use of vasoactive drugs (epinephrine, norepinephrine), ST-segment elevation, and percutaneous coronary intervention.

Two intensivists (YB and MR) independently reviewed the hospital records of each patient and categorized the reason for death as follows (adapted from Witten et al.[6]): RPRS, defined as refractory hemodynamic shock considered secondary to OHCA, including subsequent multiorgan failure, leading to death despite aggressive critical care (e.g., vasopressive or mechanical support); brain death; recurrent cardiac arrest; WLST warranted by severe hypoxic–ischemic brain injury, and WLST warranted by comorbidities.

### Statistical analysis

We described categorical variables as proportions and continuous variables as median [interquartile range]. Comparisons were performed with Pearson’s Chi-square test for categorical variables and Student’s *t*-test or Wilcoxon’s rank sum test for continuous variables. Agreement between the two investigators who determined the reason for death was assessed by computing the kappa coefficient.

Once clusters were identified, we compared differences using Pearson’s Chi-square test for categorical variables and ANOVA or Kruskal–Wallis test for continuous variables. Under the missing-at-random assumption, we imputed missing data for covariates using multiple imputations by chained equations, with logistic models for binary variables and predictive mean-matching for continuous variables. We created 20 datasets with missing values replaced by imputed values.

#### Hierarchical cluster analysis

We performed an unsupervised hierarchical cluster analysis (without reason for death among the clustering variables) based on Utstein variables including sex, age, presence of a witness, bystander CPR, location of the OHCA, first-recorded cardiac rhythm, total epinephrine dose delivered during advanced life support, no-flow and low-flow times, arterial lactate and serum creatinine at ICU admission. We sought to maximize within-cluster uniformity and to maximize differences across clusters. Hierarchical cluster analysis was used to identify the optimal number of clusters according to the minimal relative inertia loss [25]. The steps of this hierarchical cluster analysis were as follows. First, the clinical and laboratory variables were processed by dimensionality reduction using factor analysis of mixed data to correct for multicollinearity and to reduce data noise [26, 27]. We relied on the explained total inertia to determine the number of variables to include in the model. Ten dimensions (i.e., linear combinations of variables) explained 83% of the total inertia and were kept in the model. Second, to identify the optimal number of clusters, we included these ten dimensions in a hierarchical cluster analysis using Euclidean distance measures, with Ward’s linkage criterion [28] to minimize total within-cluster inertia. Third, we optimized within-cluster uniformity by using the k-means method to consolidate the optimal number of clusters. To determine the ideal number of clusters, we employed the elbow method [29, 30], identifying the point at which the decrease in within-cluster variability becomes flat. We assessed the reproductivity of the clustering using bootstrap dataset. Finally, we described the characteristics of the identified clusters by computing the *V*-test score to reflect the rank importance of each variable in each cluster. For a given cluster, a positive *V*-test score (≥ 1.96) for a variable indicates that this variable is overrepresented in the cluster compared to other clusters. By contrast, a negative *V*-test score (≤ − 1.96) indicates that the variable is underrepresented in the cluster.

#### Survival analyses

We performed survival analyses to assess the cumulative risk of RPRS in each cluster. The analysis period started at ICU admission and the time-to-event analysis was censored at the date of death or of ICU discharge alive. The proportionality assumption was assessed via log–log (survival) vs. log (time) plots. We described the incidence of RPRS in each cluster, using the Nelson–Aalen non-parametric estimator to account for competing risks. The global Gray test was applied to compare survivor function equality. In our analysis, death for reasons other than RPRS were competing risks. Fine and Gray proportional hazards regression models, accounting for competing risks [31], were built to estimate the hazard ratios (HRs) of RPRS associated with each cluster. We also performed a sensitivity analysis by applying the Kaplan–Meier method and log-rank tests to assess the significance of differences across clusters and by building Cox models to estimate the HRs.

All analyses were two-sided with a significance level of 0.05. We used RStudio version 1.4.1103 (RStudio PBC, Boston, MA) for the statistical analyses.

## Results

**Fig. 1:**
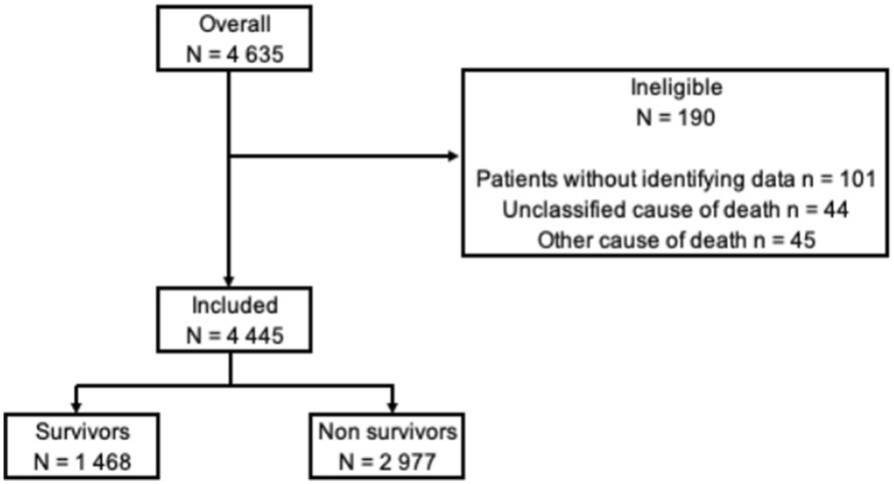
Patient flowchart

Figure 1 is the patient flowchart. Between May 15, 2011, and December 31, 2018, 4635 patients were admitted alive to ICUs after OHCA without external causes, and 4445 were enrolled in the study, including 1468 (33%) who survived to ICU discharge and 2977 who died in the ICU (67%).

### Baseline characteristics

Table 1 reports the main baseline characteristics of the 4445 study patients and compares the survivors and nonsurvivors. Overall, 528 patients treated with ECMO were included (12% of the whole population). The main reason for death in the ICU was WLST warranted by hypoxic–ischemic brain injury (1034/2977, 35%), followed by RPRS (832/2977, 28%). Brain death occurred in 481 (481/2977, 16%) patients. Agreement between investigators regarding classification of the reason for death was good, with a kappa coefficient of 0.87.

**Table 1:** Baseline characteristics of the 4445 study patients

Characteristics	Overall population *N* = 4445	Nonsurvivors^a^ *N* = 2977	Survivors^a^ *N* = 1468	*P* value^b^
Males, *n* (%)	3112 (70)	1999 (67)	1113 (76)	< 0.001
Age (years), median [IQR]	62 [51–73]	64 [53–75]	57 [48–68]	< 0.001
Witnessed, *n* (%)	4001 (90)	2591 (87)	1410 (96)	< 0.001
Bystander CPR, *n* (%)	2985 (75)	1798 (69)	1187 (85)	< 0.001
OHCA in a public area, *n* (%)	1871 (42)	1023 (34)	848 (58)	< 0.001
Shockable rhythm, *n* (%)	2217 (53)	1053 (37)	1164 (85)	< 0.001
No-flow time^c^ (min), median [IQR]	3.0 [0.0–8.0]	5.0 [0.0–10.0]	1.0 [0.0–5.0]	< 0.001
Low-flow time^d^ (min), median [IQR]	22 [14–35]	26 [17–40]	15 [10–23]	< 0.001
ST-segment elevation, *n* (%)	1602 (45)	866 (38)	736 (56)	< 0.001
Epinephrine dose (mg), median [IQR]	2.0 [0.0–4.0]	3.0 [1.0–5.0]	0.0 [0.0–1.0]	< 0.001
TTM, *n* (%)	2300 (53)	1425 (49)	875 (62)	< 0.001
Vasoactive drugs, *n* (%)	2735 (66)	2060 (75)	675 (49)	< 0.001
Successful angioplasty, *n* (%)	1201 (27)	614 (21)	587 (40)	< 0.001
Lactate (mmol/L), median [IQR]	5.8 [2.9–10.6]	7.9 [4.4–12.5]	2.9 [1.7– 4.8]	< 0.001
Creatinine (µmol/L), median [IQR]	112 [86–145]	126 [98–162]	92 [75–116]	< 0.001
Patients treated with ECMO, *n* (%)	528 (12)	442 (15)	86 (6)	<0.001

*CPR* cardiopulmonary resuscitation, *ROSC* return of spontaneous circulation, *TTM* targeted temperature management

^a^Survival was determined at discharge from the intensive care unit

^b^χ^2^ test for categorical variables, Student’s *t*-test or Wilcoxon’s rank sum test for continuous variables

^c^No-flow time was the time from collapse to the initiation of cardiopulmonary resuscitation

^d^Low-flow time was the time from the initiation of cardiopulmonary resuscitation to the return of spontaneous circulation

Additional file 1: Fig. S1: shows the times for each reason for death. Of the 832 cases of RPRS, 772 (93%) occurred within 3 days after ICU admission. In contrast, of the 1034 patients with WLST for hypoxic–ischemic brain injury, 777 (75%) died on day 4 or later

### Hierarchical cluster analysis

**Fig. 2:**
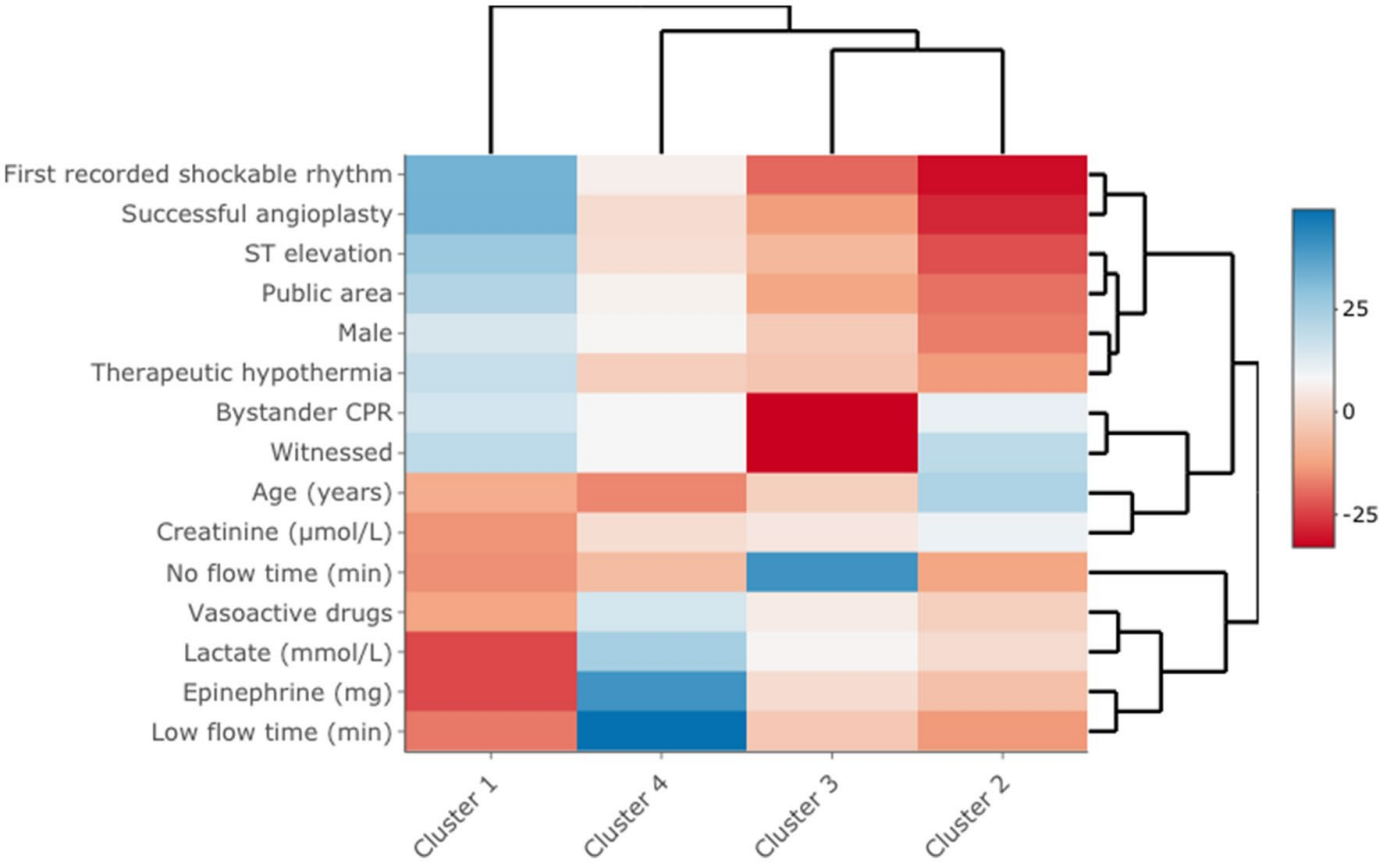
Heatmap (*v*-test score scale) of clinical and laboratory variables in each of the four clusters. A *V*-test score ≥ 1.96 or ≤ − 1.96 was taken as the cutoff indicating variable over- or underrepresentation in clusters. For example, in cluster 1, a first-recorded shockable rhythm was significantly overrepresented (*V*-test score, 33; blue color), whereas no-flow time was shorter than in the other clusters (*V*-test score, − 14.9; red color)

**Fig. 3:**
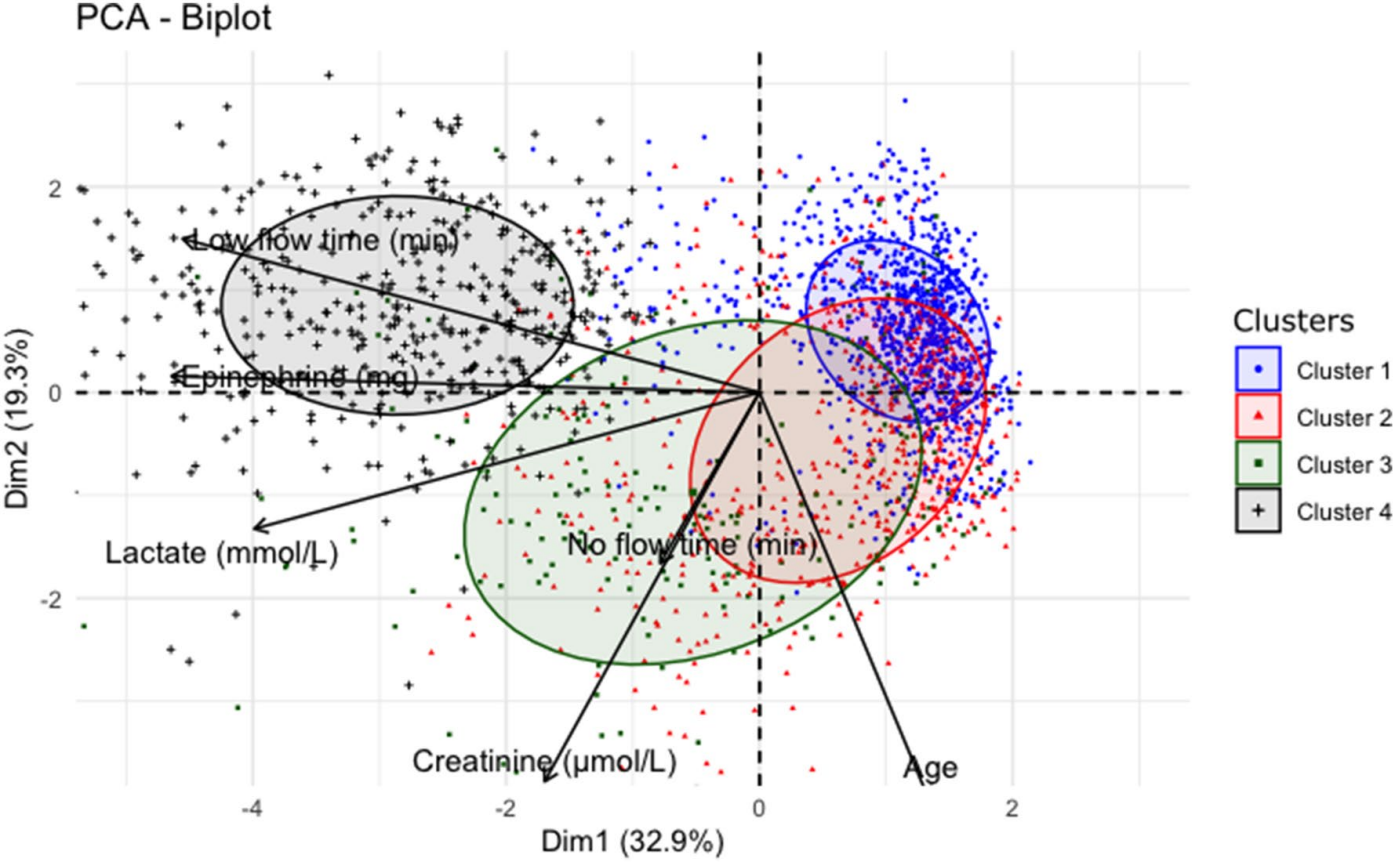
Biplot representation of clinical and laboratory variables in each of the four clusters. *PCA* principal component analysis, *Dim* dimension

Ten dimensions explained 83% of the total inertia and were used to build the model. Unsupervised hierarchical clustering identified four clusters, with 1619, 1528, 727, and 571 patients, respectively (Table 2, Fig. 2, and Additional file 2: Fig. S2). Compared to the other clusters, cluster 1 had characteristics of “male with ischemic OHCA”: larger proportions of shockable rhythms, ST-segment elevation, and a higher proportion of angioplasty. Cluster 2 included mostly “women with non-ischemic OHCA”: smaller proportions of shockable rhythms, fewer ST-segment elevation, and fewer males. Cluster 3 was characterized by “non-witnessed, delayed treated OHCA”: fewer patients had a witness and received bystander CPR, resulting in a longer no-flow time. Finally, patients in cluster 4 had “difficult to treat OHCA, with aggressive and prolonged resuscitation”: higher epinephrine doses during resuscitation, longer low-flow times, and higher serum lactate levels at ICU admission. The two-dimensional biplot representation highlights the main differences across clusters (Fig. 3). Sensitivity analysis using bootstrap showed a strong reproductivity of the clusters.

**Table 2:** Utstein clinical and laboratory features in each of the four clusters (imputed model)

Characteristics	Cluster 1 *N* = 1619	Cluster 2 *N* = 1528	Cluster 3 *N* = 727	Cluster 4 *N* = 571	*P* value^a^
Prehospital characteristics					
Males, *n* (%)	1344 (83)	822 (54)	472 (65)	474 (83)	< 0.001
Age (years), median [IQR]	58 [49–68]	69 [60–80]	61 [50–72]	53 [44–61]	< 0.001
Witnessed, *n* (%)	1615 (100)	1528 (100)	299 (41)	561 (98)	< 0.001
Bystander CPR, *n* (%)	1324 (82)	1208 (78)	6 (0.9)	466 (82)	< 0.001
OHCA in public area, *n* (%)	1032 (64)	358 (23)	167 (23)	315 (55)	< 0.001
Shockable rhythm, *n* (%)	1511 (93)	324 (21)	142 (19)	370 (65)	< 0.001
No-flow^b^ (min), median [IQR]	2 [0–5]	2 [0–5]	15 [10–20]	2 [0–5]	< 0.001
Low-flow^c^ (min), median [IQR]	18 [10–26]	20 [13–30]	25 [15–34]	80 [52–100]	< 0.001
ST-segment elevation, *n* (%)	1149 (71)	334 (22)	227 (31)	278 (49)	< 0.001
Epinephrine dose (mg) Median [IQR]	0 [0–2]	2 [1–4]	3 [1–4]	8 [5–11]	< 0.001
Hospital management					
TTM, *n* (%)	1142 (71)	601 (39)	335 (46)	280 (49)	< 0.001
Vasoactive drugs, *n* (%)	902 (56)	986 (65)	552 (76)	525 (92)	< 0.001
Angioplasty success, *n* (%)	933 (58)	69 (4.5)	68 (9.4)	176 (31)	< 0.001
Lactate (mmol/L), median [IQR]	3.4 [1.9–5.7]	6.5 [3.4– 11.0]	8.1 [4.2– 13.0]	12.4 [8.5– 17.0]	< 0.001
Creatinine (µmol/L), median [IQR]	97 [78–121]	124 [93–170]	124 [93–164]	132 [105–160]	< 0.001
Patients treated with ECMO, *n* (%)	107 (7)	39 (3)	23 (3)	359 (63)	< 0.001
Follow-up (days), median [IQR]	6 [3–10]	2 [1–7]	2 [1–6]	1 [0–4]	< 0.001

*CPR* cardiopulmonary resuscitation, *TTM* targeted temperature management

^a^*χ*^2^ test for categorical variables, ANOVA or Kruskal–Wallis test for continuous variables

^b^No-flow was the time from collapse to the initiation of cardiopulmonary resuscitation

^c^Low-flow was the time from the initiation of cardiopulmonary resuscitation to the return of spontaneous circulation

**Fig. 4:**
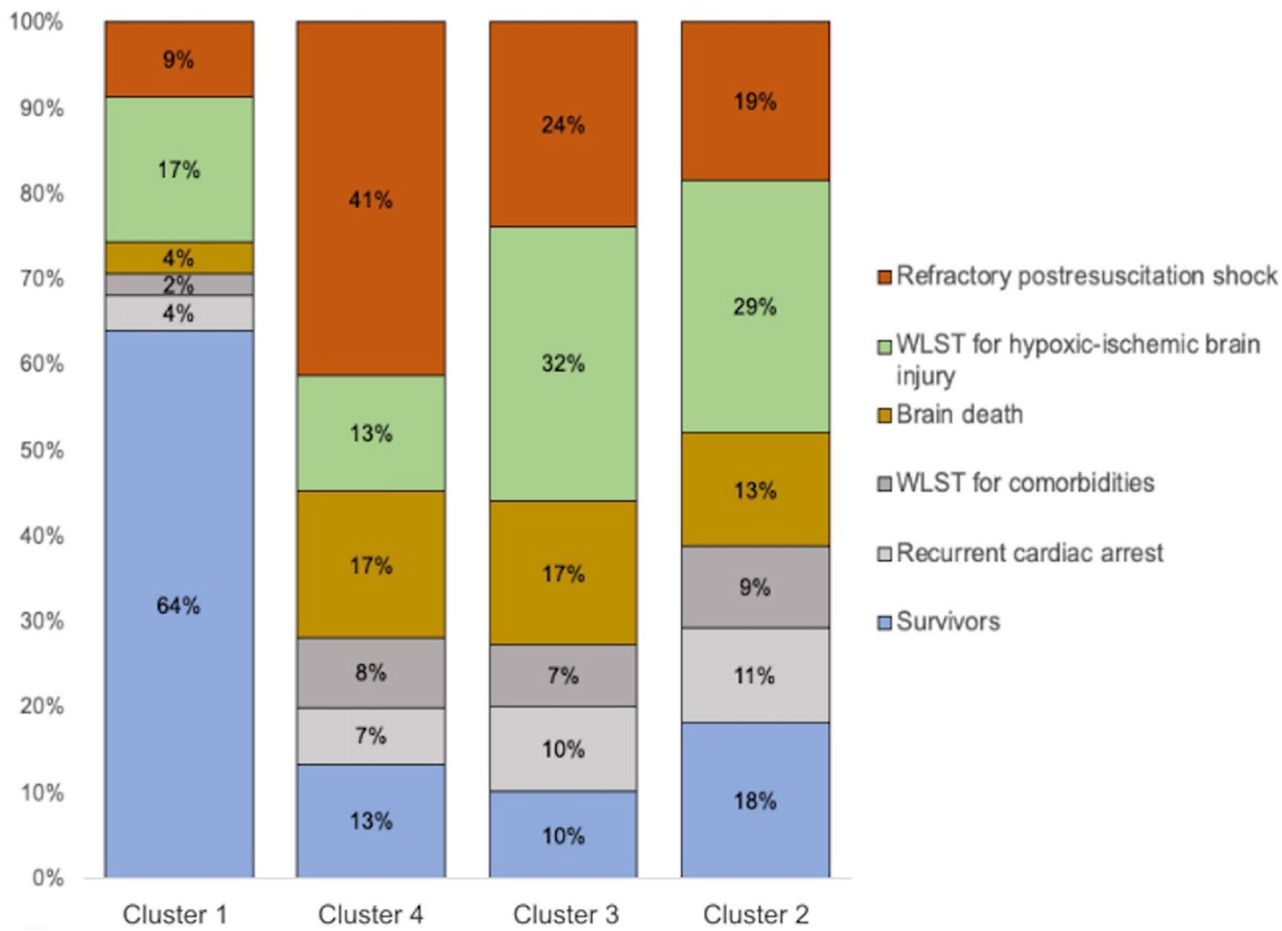
Vital status and reasons for death in each of the four clusters. *WLST* withdrawal of life-sustaining treatments

Vital status and reason for death differed significantly across clusters (Fig. 4). Survival was significantly higher in cluster 1 (1036/1619, 64%) than in each of the other three clusters (*P* < 0.01 for all comparisons). RPRS was significantly more common in cluster 4 (237/571, 41%) than in each of the other clusters (*P* < 0.01 for all comparisons).

### Survival analysis

**Fig. 5:**
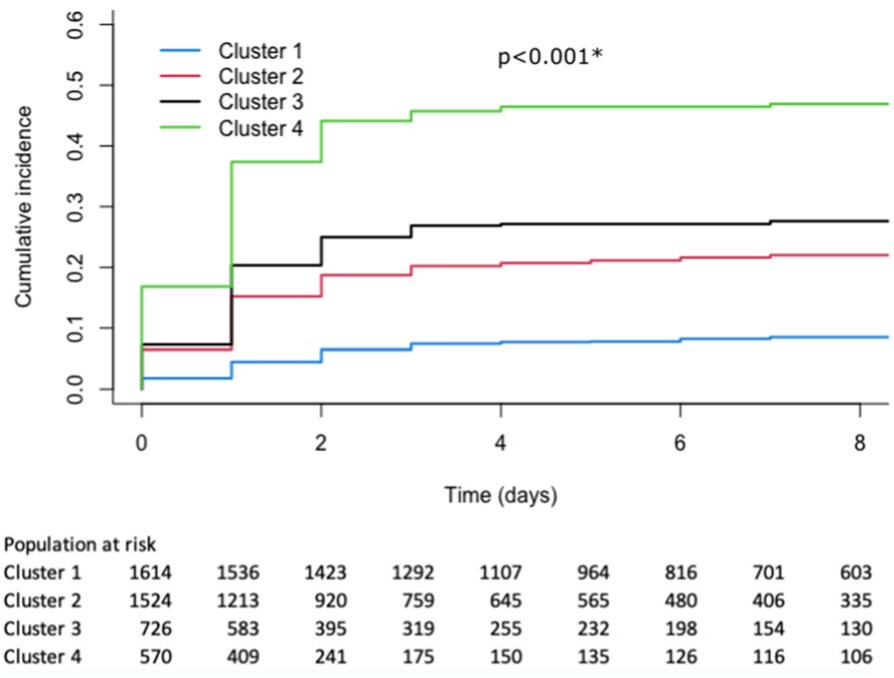
Cumulative incidence of refractory postresuscitation shock (RPRS) in each of the four clusters

Median follow-up was 6 days [4–12 days] in survivors and 2 days [1–6 days] in nonsurvivors. The unadjusted cumulative hazard functions for RPRS differed significantly across clusters (*P* < 0.001, global Gray test) (Fig. 5). The incidence of RPRS was significantly higher in cluster 4 compared to each of the other clusters (*P* < 0.001 for all comparisons) and significantly lower in cluster 1 compared to each of the other clusters (Table 3). The sensitivity analysis done using Kaplan–Meier and Cox models showed similar results with a significant difference across clusters (*P* < 0.001, global log-rank test) and a significant association between cluster 4 and RPRS (HR, 3.12; 95% CI 2.35–4.14).

**Table 3:** Hazard ratios (Fine and Gray) for refractory postresuscitation shock associated with each cluster

	Events^a^	HR	95%CI	*P* value
Cluster 1	140	Reference	–	
Cluster 2	284	2.20	1.80–2.68	< 0.001
Cluster 3	171	2.82	2.26–3.50	< 0.001
Cluster 4	237	5.51	4.51–6.74	< 0.001

*HR* hazard ratio, *95%CI* 95% confidence interval

^a^Deaths due to refractory postresuscitation shock

## Discussion

Our population-based, real-life study utilized an innovative, unsupervised clustering analysis that yielded several important findings. Firstly, two-thirds of patients admitted to the ICU after OHCA died before ICU discharge, with RPRS accounting for 28% of these deaths. Secondly, we identified four distinct clusters based on their baseline characteristics. Notably, one of these clusters was characterized by longer low-flow times, higher epinephrine doses and higher lactate levels. Finally, patients in this cluster were found to be at a significantly higher risk of RPRS, indicating the potential importance of targeted interventions aimed at this mode of death.

In our large population-based registry, among patients admitted alive after OHCA, ICU mortality was 67%. This result is consistent with previous studies (61% [4], 66% [5], and 57% [32]) and with a 2020 meta-analysis [33]. To strengthen our analysis, we used a previously published classification to characterize the reasons for death [6] and adjudication of the reason for death was performed by two independent investigators. Regarding mode of death, our population is in line with previous data, the most common reasons (51%) being neurological death (brain death or WLST for hypoxic–ischemic brain injury) [5, 6, 34]. We also evaluated the time to death in the ICU for each reason. There again, our data confirmed previously published populations, with over 90% of deaths from RPRS occurring during the first 3 days [5]. Overall, our findings in a large population provide external validation of previous data, and mortality, mode and timing of deaths of our patients are highly similar with other studies, reinforcing the external validity of our results. The adjudication of the reason for death by two independent investigators is among the strengths of our study.

RPRS is a complication of postresuscitation disease, which is due to both the underlying cause of OHCA and ischemia–reperfusion syndrome [19, 35–37]. Postresuscitation disease can combine myocardial dysfunction [38–40], vasoplegia [41–43], and relative adrenal insufficiency [42, 44]. Interventions being evaluated for preventing RPRS include steroids [7–10], ciclosporine [11], and goal-directed hemodynamic optimization [14–16]. These interventions have not been proven beneficial in unselected populations but have not been evaluated in patient subgroups defined by their risk of specific adverse outcomes. Two previous studies looked for factors associated with a higher risk of circulatory death. In a multicenter cohort of 956 patients, a model based on five factors had an area under the receiver-operating-characteristics curve of 0.73 for predicting RPRS [32]; and in a single-center study of 303 patients, arterial pH below 7.11 and need for vasoactive drugs at ICU admission were associated with RPRS [45]. In contrast to previous studies, which utilized a smaller set of variables and a priori selection of variables, our analysis included 10 dimensions (linear combination of variables) and a larger set of variables in an unselected population. Our data-driven approach enabled an agnostic exploration of the data, and we performed a centralized, double-adjudicated mode of death, which is a significant strength compared to the two studies cited. This rigorous and comprehensive approach provides greater confidence in the accuracy and reliability of our findings. The cluster 4 had specific features that might prove helpful in selecting patients and designing post hoc analyses of treatments targeting RPRS (for example, arginine-vasopressin and/or hydrocortisone, NCT04591990). Ideally, clustering of patients should allow for the identification of RCT candidates as early as possible after ROSC. However, our methodology requires information on interventions such as angioplasty or hypothermia that are only available after ICU admission.

Cluster 1 had large proportions of patients with first-recorded shockable rhythms, angioplasty, with shorter no-flow and low-flow times at ICU admission. Consistent with these favorable characteristics, survival was highest and the frequency of RPRS lowest in this cluster. Patients in cluster 2 rarely had a first-recorded shockable rhythm or ST-segment elevation and had short no-flow times, contrasting with the long no-flow times in cluster 3. Most patients in these two clusters (2 and 3) died after WLST warranted by hypoxic–ischemic brain injury. They might constitute the population most likely to benefit from neuroprotective treatments such as targeted temperature management.

Our study has several strengths. To the best of our knowledge, it is the largest in its field. The reason for death was adjudicated centrally by two investigators working independently from each other. Moreover, interobserver agreement was good (Kappa 0.87, compared to 0.61 in a previous study [6]). Competing risks are a major issue in studies of OHCA and we accounted for them by using a Fine and Gray model. Our population was composed of consecutive unselected patients managed at multiple centers in the real-life setting. Finally, we conducted an unsupervised clustering analysis to distinguish patient subgroups, thereby acquiring additional information over that provided by studies of overall mortality.

The limitations of our study include missing data for some of the variables. Nonetheless, we performed multiple imputation to circumvent this issue. We were unable to include echocardiographic parameters among the variables used to characterize patients and clusters. However, confining the study to variables immediately available at ICU admission, even to clinicians without echocardiography skills, can also be seen as an advantage. Furthermore, the multicentric nature of our registry presents a potential risk of bias. There is a possibility of different definitions of RPRS (which could lead to outcome detection bias) and variations in the timing of lactate measurement or ICU treatment strategies due to local policies. However, most of the variables included in our analysis are independent of local practices, and we believe that the advantages provided by the multicentric design outweigh the potential disadvantages. We cannot exclude residual confounding by unmeasured factors. Firstly, we were unable to collect data on comorbidities or past medical history of patients, which could have been useful for clustering analysis, but were not available in our database, potentially leading to information bias. Secondly, due to limited availability, echocardiographic parameters could not be included in our characterization of patients and clusters. Finally, although lactate and creatinine were included as biological markers, some data such as pH were not available. Nevertheless, the dimensions used to build our model explained 83% of the total inertia. Further prospective work is needed to assess our cluster analysis results.

## Conclusion

In this population-based unsupervised clustering analysis of over 4400 patients, we identified a specific subpopulation at high risk for death from RPRS. These patients might be most likely to benefit from interventions targeting shock.

## Supplementary Information

**Additional file 1: Fig. S1.** Times of deaths due to the five reasons, in days since admission to the intensive care unit.

**Additional file 2: Fig. S2.** Hierarchical clustering.

## Data Availability

The datasets used and/or analyzed during the current study are available from the corresponding author on reasonable request.

## Abbreviations

CPR: Cardiopulmonary resuscitation
ICU: Intensive care unit
IQR: Interquartile range
HR: Hazard ratio
OHCA: Out-of-hospital cardiac arrest
RPRS: Refractory postresuscitation shock
ROSC: Return of spontaneous circulation
WLST: Withdrawal of Life-Sustaining Treatments
