# Supplementary figures and images for: Factors associated with circulatory death after out-of-hospital cardiac arrest: a population-based cluster analysis
Source: Ann Intensive Care. 2023 Jun 9;13:49. doi: 10.1186/s13613-023-01143-8 (PMC10256665; doi:10.1186/s13613-023-01143-8)

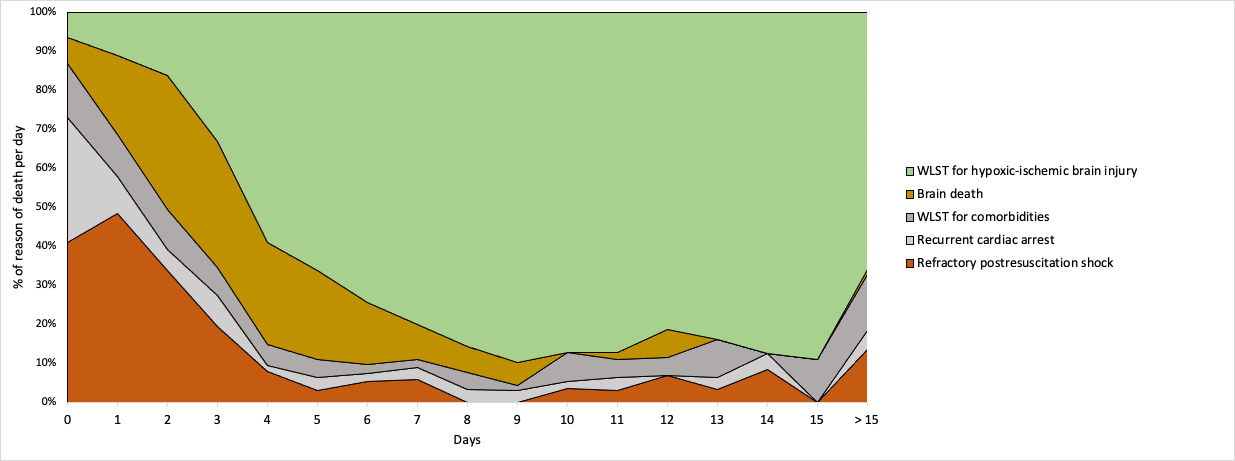

Supplement: Supplementary file 1 — Additional file 1: Fig. S1. Times of deaths due to the five reasons, in days since admission to the intensive care unit. [file 13613_2023_1143_MOESM1_ESM.tiff]

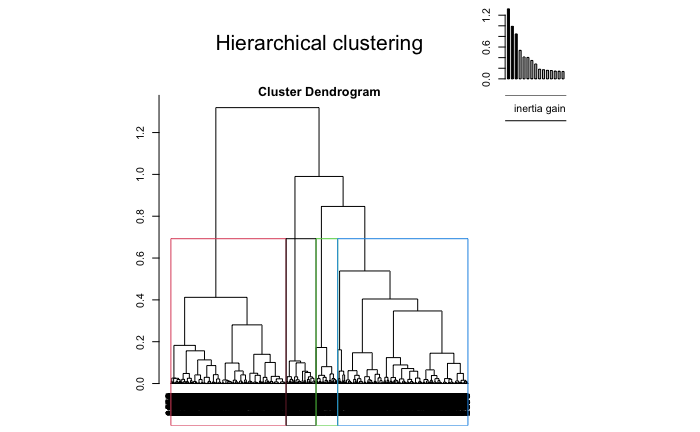

Supplement: Supplementary file 2 — Additional file 2: Fig. S2. Hierarchical clustering. [file 13613_2023_1143_MOESM2_ESM.tiff]
